# Change in renin, cardiovascular and inflammatory markers over three years in a black and white population: the SABPA study

**DOI:** 10.1186/s12872-017-0538-x

**Published:** 2017-04-26

**Authors:** Rijané Swart, Johannes M. van Rooyen, Catharina M. C. Mels

**Affiliations:** Hypertension in Africa Research Team (HART), North-West University (Potchefstroom Campus), Private Bag X6001, Potchefstroom, 2522 South Africa

**Keywords:** Race, C-reactive protein, Hypertension, Inflammation, Interleukin-6, Low renin, Systolic blood pressure

## Abstract

**Background:**

To investigate if percentage change (%∆) in renin over a 3 year follow-up is associated with %∆ in cardiovascular and inflammatory markers in a low renin bi-ethnic group.

**Methods:**

Blood pressure, active renin, C-reactive protein and interleukin-6 levels of 73 black and 81 white teachers were measured at baseline and after 3 years.

**Results:**

In the black group, %∆ renin was inversely associated with %∆ systolic blood pressure (β = −0.27; *p* = 0.011). In the white group %∆ renin was inversely associated with %∆interleukin-6 (β = −0.24; *p* = 0.005).

**Conclusions:**

These prospective results indicate that a decrease in renin over time is associated with an increase in blood pressure in a low renin black South African cohort.

**Electronic supplementary material:**

The online version of this article (doi:10.1186/s12872-017-0538-x) contains supplementary material, which is available to authorized users.

## Background

The prevalence of hypertension in South Africa is a major concern. A health survey done in 2001 stated that 8.2 million people above the age of 15 years had hypertension [1]. Furthermore, only 2.7 million people with hypertension receive anti-hypertensive treatment [1]. More recently it was indicated that 78% of South Africans above 50 years of age have hypertension, underlining the increasing burden of cardiovascular disease (CVD) in South Africa [2].

Increased renin, the rate limiting step in the renin-angiotensin system (RAS), is associated with increased synthesis of angiotensin II (Ang II) from angiotensinogen [3]. In turn, increased Ang II levels are associated with increased inflammation [4] and may lead to the development of hypertension [5]. On the other hand, suppressed RAS activity, as a consequence of low renin levels, may lead to the development of volume loading hypertension (low-renin hypertension) [6].

Lower renin levels in black participants when compared to their white counterparts were previously reported [7, 8]. Additionally, we also indicated inverse associations of renin levels with ambulatory systolic and diastolic blood pressure in black men [9]. In low renin black men and women, renin was also associated with the albumin-to-creatinine ratio, suggesting a link between low renin levels and subclinical end-organ damage [10].

In contrast with the lower renin activity found in black participants, levels of inflammation are higher in black participants when compared to white participants [11]. We previously linked increased blood pressure with increased inflammation in a high C-reactive protein (CRP) (>3 mg/L) group of black men [12]. In the present sub-study we therefore aimed to investigate if change (over a three-year period) in renin levels is associated with change in cardiovascular and inflammatory markers in a low renin black and white cohort.

## Methods

### Study design and population

This study formed part of the Sympathetic activity and Ambulatory Blood Pressure in Africans (SABPA) prospective cohort study that took place between 2008/9 (baseline) and 2011/12 (follow-up). A detailed description of the study population and protocol has been published [13]. At baseline 409 black and white teachers with a similar sex distribution were included and 359 teachers returned for the follow-up study (87.3% compliance to follow-up). Exclusion criteria at baseline included the use of α- and β-blockers, an ear temperature > 37.5 °C, participants who were vaccinated or who donated blood 3 months prior to the commencement of the study and pregnant or lactating women. For this sub-study we also excluded participants who did not participate in both the baseline and follow-up phases (*n* = 50) and participants with no renin values at baseline (*n* = 35) and follow-up (*n* = 68). Additionally, in accordance with our aims participants with a renin value of ≥6.18 pg/ml at baseline (*n* = 88) and follow-up (*n* = 17) was excluded in this study. The cut-off value of 6.18 pg/ml was selected based on the mean renin value in healthy subjects between 40 and 60 years of age (supine range:1.1-20.2 pg/ml) (Renin III Generation, CIS Biointernational, Cedex, France) [10]. HIV infected participants (*n* = 13) and participants on medication (*n* = 1) that could interfere with the RAS was also excluded. After applying these exclusion criteria our baseline study population consisted of 73 black and 81 white participants while data from 66 black and 71 white participants were used to investigate change over a 3 year period.

### Anthropometric measurements

Weight and height of the subjects were taken in triplicate with calibrated instruments (Precision Health Scale, A & D Company, Tokyo, Japan and Invicta Stadiometer, IP 1465, UK) and body mass index was calculated [14].

### Questionnaires

All the subjects completed a general health questionnaire containing questions on their lifestyle habits such as smoking and medication usage.

### Cardiovascular measurements

The validated Finometer device [15, 16] was used to measure systolic blood pressure (SBP) and diastolic blood pressure (DBP) after 5 min resting period (Finapres Medical Systems, Amsterdam, The Netherlands). Total peripheral resistance (TPR) and Windkessel compliance (Cwk) were calculated using the Fast Modelflo computer software program from the Finometer data. The Sonosite Micromaxx ultrasound system (SonoSite Inc., Bothell, WA) and a 6-13 MHz linear array transducer was used to measure carotid intima media thickness (cIMT), by obtaining a high resolution image from two angles from the left and right carotid artery. A 10 mm segment with good image quality was chosen after the images were imported into the Artery Measurement System (AMS) II v1.139 (Gothenburg, Sweden). The mean cIMT were calculated through determining the borders of the far and near wall and the inner diameter of the vessel from 100 discrete measurements through the 10 mm segment for each participant. The analysis of the cIMT was done by one observer and the intra-observer variability was 0.04 mm between two measurements made 4 weeks apart (*n* = 10).

### Biochemical analyses

Experienced nurses obtained blood samples from fasting participants from brachial antecubital vein branches with a sterile winged infusion set. Blood was drawn from participants when in a supine position. Serum, ethylenediaminetetraacetic acid (EDTA) and sodium fluoride plasma were prepared according to standard procedures and stored at −80 °C until biochemical analyses were performed. Active renin was determined with a radio-immunometric assay (Renin III Generation, CIS biointernational, Cedex, France) in EDTA plasma. Serum IL-6 levels were measured using a high sensitivity Quantikine enzyme linked immunosorbent assay (ELISA) (R&D Systems, Minneapolis, MN USA). High sensitivity CRP, gamma glutamyl transferase (GGT) as measure of alcohol abuse [17], triglycerides and total cholesterol levels were determined in serum while glucose levels were determined in sodium fluoride plasma with three multiple analysers (Cobas Integra 400 plus, Roche, Basel, Switzerland; Konelab 20i; Thermo Scientific, Vantaa, Finland and Unicel DXC 800, Beckman and Coulter, Germany). The intra- and inter-coefficients of variation for all assays were below 10%. Von Willebrand Factor (vWf) was determined with an ELISA assay in which a polyclonal rabbit anti-vWf antibody and rabbit anti-vWf-horse radish peroxidase (HRP) antibody (DAKO, South Africa) were used. Serum cotinine levels were measured using a homogeneous immunoassay (Automated Modular, Roche, Basel, Switzerland).

### Statistical analyses

Statistical analyses were performed with Statistica 12 (Statsoft Inc., Tulsa, OK, USA). Values are regarded as significant when *p* ≤ 0.05. Data were expressed as arithmetic mean and standard deviation for normally distributed variables. Variables with a non-Gaussian distribution (renin, IL-6, CRP, vWf, cotinine, GGT) were logarithmically transformed and the central tendency and spread represented by the geometric mean and the 5th and 95th percentile intervals. Means and proportions were compared between black and white groups using independent t-tests and Chi-square tests, respectively. Proportions were compared at baseline and follow-up with the McNemar tests, in black and white low renin groups. Percentage change for all variables were calculated and compared between black and white groups while adjusting for age, sex and baseline values using analyses of covariance (ANCOVA). Correlations of percentage change in renin with percentage change in biochemical and cardiovascular variables were performed using partial regression analyses (adjusted for sex and age at baseline). Forward stepwise multiple regression analyses were performed to determine independent associations of percentage change in renin with percentage change in inflammatory and cardiovascular variables. Two models were compiled with percentage change in renin as the dependent variable in both models. In model 1 the main independent variable was percentage change in SBP. The covariates that were entered in model 1 were age, baseline SBP, baseline renin, percentage change in IL-6, baseline IL-6, baseline GGT and percentage change in GGT. We selected covariates for inclusion in our models based on the literature. Age, since blood pressure and vascular change is dependent of age [18], and the black population is known to experience early vascular ageing it was added as covariate to our models. Baseline SBP, baseline renin, baseline IL-6 and baseline GGT, because we used the percentage change of these variables in the model. Percentage change in IL-6, since our aim was to investigate if change (over a three-year period) in renin levels is associated with change in inflammatory markers thus IL-6. Increased IL-6 may indicate future risk for CVD [19]. Percentage change in GGT, because GGT is a marker of alcohol usage, it may have a strong influence on hypertension and inflammation [20].

In model 2 the main independent variable was percentage change in PP. The covariates entered for model 2 were age, baseline PP, baseline renin, percentage change in IL-6, baseline IL-6, baseline GGT and percentage change in GGT. In a post hoc analyses the achieved power (1-β) were determined for multiple regression models with 8 variables, for a group with a sample size of *n* = 76 for blacks (1-β = 0.999) and *n* = 99 for whites (1-β = 0.77) (G*power v3.1.9.2) [21]. The effect sizes (f2) were determined from the partial regression coefficient values and the significance level (α) was set at 0.05. In sensitivity analyses, we substituted percentage change in IL-6 with percentage change in CRP and age with BMI in separate analyses. Since anti-inflammatory medication usage may influence the inflammatory response and the results, we also added anti-inflammatory medication usage as covariate and repeated the multiple regression analyses.

## Results

The characteristics of black (*n* = 73) and white (*n* = 81) participants with low renin at baseline are compared in Table 1. The cardiovascular profile of black and white participants with low renin was comparable, except for the higher DBP (*p* = 0.02) noted in the black group compared to the white group. The number of participants who were hypertensive increased from baseline to follow-up, black group: *n* = 18 (27.3%) to *n* = 27 (40.91%) (*p* = 0.087) and white group: *n* = 8 (11.27%) to *n* = 15 (21.13%) (*p* < 0.001). In both the low renin black and white groups the number of participants using anti-hypertensive medication increased from baseline to follow-up, black group: *n* = 0 (0.00%) to *n* = 12 (18.2%) (*p* < 0.001) and white group: *n* = 1 (1.14%) to *n* = 8 (11.3%) (*p* < 0.001).

**Table 1:** Characteristics of low renin black and white participants at baseline

	Low renin		
	Black (*n* = 73)	White (*n* = 81)	*p*-value
Age (years)	41.2 ± 6.55	46.9 ± 8.72	<0.001
Sex (n, male)	36	31	0.167
Body mass index (kg/m^2^)	28.1 ± 5.86	27.0 ± 5.44	0.232
Cardiovascular measurements			
Systolic blood pressure (mmHg)	136 ± 16	135 ± 14	0.793
Diastolic blood pressure (mmHg)	79 ± 9	76 ± 7	0.021
Pulse pressure (mmHg)	56.4 ± 10.4	58.9 ± 10.7	0.141
Total peripheral resistance (mmHg/ml/s)	1.01 ± 0.25	1.05 ± 0.3	0.399
Windkessel compliance (ml/mmHg)	1.91 ± 0.40	2.02 ± 0.54	0.148
Carotid intima media thickness (mm)	0.65 ± 0.10	0.66 ± 0.11	0.557
Biochemical analyses			
Renin (pg/ml)	3.87 (2.21;6.66)	4.59 (2.43;6.98)	0.002
Interleukin-6 (pg/ml)	2.16 (2.06;2.22)	2.15 (2.08;2.22)	0.286
C-reactive protein (mg/l)	5.07 (2.27;26.6)	4.12 (2.99;11.0)	0.006
Glucose (mmol/l)	5.15 ± 0.92	5.59 ± 0.71	0.001
Von Willebrand factor (%)	86.2 (54.9;135)	60.1 (40.9;87.1)	<0.001
Triglycerides (mmol/l)	1.18 ± 1.03	1.16 ± 0.69	0.882
Total cholesterol (mmol/l)	4.48 ± 1.17	5.84 ± 1.19	<0.001
Lifestyle			
γ-Glutamyltransferase (U/l)	36.7 (16.6;180)	16.4 (7.00;76.0)	<0.001
Cotinine (ng/ml)	2.58 (2.00;114)	2.58 (2.0;245)	0.669
Smoking (n)	8	14	0.249

Data expressed as mean ± standard deviation, geometric mean (5th and 95th percentiles) and n

The black group had lower renin (3.87 pg/ml vs. 4.59 pg/ml; *p* = 0.002), glucose (*p* = 0.001) and total cholesterol (*p* < 0.001) levels. The inflammatory marker, CRP (*p* = 0.006) as well as vWf levels (*p* < 0.001) were higher in the black participants when compared to their white counterparts. Both cotinine and self-reported smoking were similar between the black and white groups, but the black participants had higher GGT levels (*p* < 0.001) compared to the white participants.

In Table 2, the percentage change in the anthropometric, cardiovascular, lifestyle and biochemical variables are shown. Both SBP and DBP increased in the black group, while it decreased in the white group, with the difference between the groups being significant (SBP: *p* = 0.02 and DBP: *p* = 0.059). Although TPR increased in both groups, the increase in the black group was significantly higher (*p* = 0.036). The cIMT also increased in both racial groups, but to a greater extent in the whites (*p* < 0.001).

**Table 2:** Percentage change in anthropometric, cardiovascular, lifestyle and biochemical variables

	Low renin		
	Blacks (*n* = 66)	Whites (*n* = 71)	*p*-value
Body mass index (%)	4.29 (2.48;6.10)	3.35 (1.60;5.12)	0.484
Cardiovascular measurements			
Systolic blood pressure (%)	1.91 (−0.21;4.04)	−1.77(−3.84;0.30)	0.020
Diastolic blood pressure (%)	2.53 (0.29;4.77)	−0.62 (−2.80;1.56)	0.059
Pulse pressure (%)	2.43 (−1.09;5.96)	−2.52 (−5.95;0.91)	0.057
Total peripheral resistance (%)	13.0 (6.06;19.99)	2.23 (−4.55;9.01)	0.036
Windkessel compliance (%)	−3.46 (−7.68;0.76)	0.97 (−3.14;5.09)	0.169
Carotid intima media thickness (%)	1.69 (−0.73;4.12)	7.96 (5.67;10.24)	<0.001
Biochemical analyses			
Renin (%)	−17.5 (−24.3;-10.7)	2.17 (−4.39;8.74)	<0.001
Interleukin-6 (%)	59.4 (52.0;66.9)	35.5 (28.3;42.7)	<0.001
C-reactive protein (%)	0.34 (−6.80;7.48)	−9.36 (−16.3;-2.45)	0.069
Glucose (%)	0.57 (−4.73;5.87)	−22.7 (−27.9;-17.6)	<0.001
Von Willebrand factor (%)	3.11 (1.45;4.77)	5.76 (4.14;7.38)	0.048
Lifestyle			
γ-Glutamyltransferase (%)	−1.62 (−6.73;3.49)	−4.17 (−9.10;0.75)	0.512
Cotinine (%)	−1.80 (−17.2;13.6)	6.86 (−8.01;21.7)	0.443

Data expressed as adjusted mean (least square mean) percentage change with 95% confidence intervals. Adjusted for baseline age, sex and baseline values

Renin decreased in the black group and increased in the white group (−17.47% vs. 2.17%; *p* < 0.001). Regarding the inflammatory markers, IL-6 increased in both groups but more in the black group (59.44% vs. 32.51%; *p* < 0.001) while CRP increased slightly in the black group and decreased in the white group, with the difference between the groups being borderline significant (*p* = 0.069).

**Fig. 1:**
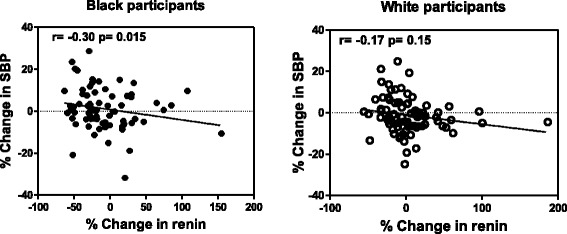
Single regression analyses of percentage change in renin and percentage change in SBP in low renin black and white groups

In single regression (Fig. 1) and partial regression analyses (Additional file 1: Table S1) percentage change in renin were inversely correlated with percentage change in SBP (*r* = −0.30; *p* = 0.015) (Fig. 1) and with percentage change in PP (*r* = −0.36; *p* = 0.003) (Additional file 1: Table S1) in the low renin black group only. These associations were absent in the low renin white group, however percentage change in renin were inversely correlated with percentage change in IL-6 (*r* = −0.25, *p* = 0.037), in the low renin white group.

In multiple regression analyses, the inverse association of percentage change in renin with percentage change in SBP in the low renin black group was confirmed to be independent of various covariates (β = −0.27; *p* = 0.011; Table 3). Additionally, percentage change in GGT (β = 0.26; *p* = 0.017) was also positively associated with percentage change in renin in the low renin black participants (model 1). In the same group, when we investigated whether the association of percentage change in renin with percentage change in PP is independent, percentage change in PP no longer entered the model (model 2). In the low renin white group the association of percentage change in renin with percentage change in IL-6 (β = −0.24; *p* = 0.005; Table 3) was also confirmed to be independent of various covariates. Power calculations indicated the population size to be sufficient.

**Table 3:** Forward stepwise regression analysis with percentage change in renin as dependent variable in black and white participants

	Percentage change in renin			
	Blacks (*n* = 76)		Whites (*n* = 99)	
Adjusted R^2^	0.34		0.54	
	β (95% CI)	*p*-value	β (95% CI)	*p*-value
Age (baseline)	0.21 (0.01; 0.41)	0.046	-	-
Systolic blood pressure (% change)	−0.27 (−0.48; −0.07)	0.011	-	-
Gamma glutamyltransferase (% change)	0.26 (0.05; 0.46)	0.017	-	-
Renin (baseline)	−0.46 (−0.66; −0.25)	<0.001	−0.71 (−0.87; −0.55)	<0.001
Interleukin-6 (% change)	-	-	−0.24 (−0.40; −0.08)	0.005

Independent variables included in the model: percentage change in SBP, baseline SBP, age, percentage change in GGT, baseline GGT, baseline renin, percentage change in IL-6 and baseline IL-6. -, Covariate did not enter model

In sensitivity analyses, we substituted percentage change in IL-6 with percentage change in CRP, the results remained the same in the low renin black group, but in the low renin white group only baseline renin (β = −0.70; *p* < 0.001) remained independently associated with percentage change in renin. When we substituted age with BMI in model 1, the results remained the same in both groups. After the addition of anti-inflammatory medication usage as covariate to the models the results also remained largely the same.

## Discussion

The aim of the study was to investigate associations of change in renin with cardiovascular and inflammatory markers in a low renin bi-ethnic South African population. The most prominent result was an independent association between a decrease in renin levels and increased SBP in black participants with low renin levels. These findings are not only in line with previous findings indicating an association between low renin and blood pressure [22] but also strengthen the suggestion that low renin may be prospectively related to the early changes in the cardiovascular system before the onset of hypertension in black South Africans [23].

There are conflicting results in the literature about the different mechanisms leading to low renin levels in black participants when compared to white participants. These factors include high renal sodium reabsorption, [23, 24] reduced renin secretion rate [25] through decreased conversion of pro-renin to renin as a result of lower soluble plasma renin receptor found in blacks [26]. Other factors include polymorphisms in genes encoding for RAS proteins [23] and lifestyle factors such as rapid urbanization [23].

Although the exact mechanism linking decreased renin levels with increased blood pressure is not clear, it may be a result of volume loading. Volume loading is associated with increased sodium retention, which may result in the retention of water by the kidneys. When water is retained it may lead to expansion of the extracellular fluid volume and thereby increasing blood pressure [6]. In turn, this may lead to an even further suppression of renin secretion [6]. Our results, furthermore, suggest that the development of volume loading hypertension may be aggravated in the black population through excessive alcohol consumption [27] as indicated by the association of percentage change in renin with GGT levels, although not a focus of this article.

In the low renin white group, percentage change in renin was negatively associated with percentage change in IL-6 (inflammation). This is difficult to explain since activation of the RAS, through an increase in the rate limiting step, (renin) may lead to increased inflammation, since increased Ang II may stimulate the production of inflammatory cytokines in vascular smooth muscle and endothelial cells [28]. Inflammatory markers (IL-6 and CRP) are reliable biomarkers related to future risk for the development of CVD [19, 29]. Although there was a slight increase (2%) in renin levels in the white group renin levels were still in the low range and may therefore not have been sufficient to elicit an increased inflammatory response.

Since there was no association between percentage change in renin levels and inflammatory markers in the low renin black group, despite the increase in inflammatory markers at follow-up. It is, therefore, expected in the black individuals that with decreased RAS activation, decreased secretion of inflammatory cytokines will ensue. However, in our study, mean IL-6 levels at baseline in the low renin black participants indicated high inflammatory levels. Our results suggest that other mechanisms may be involved in the stimulation of inflammatory responses, in this group. These mechanisms may include other enzymes such as chymase and catepsin which are involved in the conversion of Ang I to Ang II [30].

The results of this study have to be interpreted within the context of its strengths and limitations. This is a well-controlled study which included urbanized black and white participants, allowing comparison between these groups. This is, to the best of our knowledge, the first study investigating how changes in renin over a period of 3 years are associated with change in cardiovascular variables and inflammation in both black and white individuals. The limitation of this study is that our sample was relatively small and was recruited from urban areas in the Potchefstroom District of the North-West Province and cannot be seen as representative of the entire South African population. Although we investigated changes over time (3 year follow-up) our results are based on associations and we cannot infer causality. Another limitation is the unknown salt intake levels of these participants.

## Conclusion

In the black participants with low renin levels at baseline, a further decrease in renin levels over 3 years is independently associated with an increase in SBP. These results strengthen the notion that low renin may be a causative factor in the development of hypertension in black South Africans, but that the renin- angiotensin system is not the driving force behind the increased inflammation observed in this low renin black group.

## Additional file

Additional file 1: Table S1. Partial correlations of percentage change in renin with biochemical and cardiovascular variables. (DOCX 26 kb)

## Abbreviations

%∆: Percentage change
Ang II: Angiotensin
cIMT: Carotid intima media thickness
CRP: C-reactive protein
CVD: Cardiovascular disease
Cwk: Windkessel compliance
DBP: Diastolic blood pressure
GGT: Gamma glutamyl transferase
IL-6: Interleukin-6
SABPA: Sympathetic activity and Ambulatory Blood Pressure in Africans
SBP: Systolic blood pressure
TPR: Total peripheral resistance
vWf: Von Willebrand Factor
