# Supplementary material for: Change in renin, cardiovascular and inflammatory markers over three years in a black and white population: the SABPA study
Source: BMC Cardiovasc Disord. 2017 Apr 26;17:104. doi: 10.1186/s12872-017-0538-x (PMC5406936; doi:10.1186/s12872-017-0538-x)
Supplement: Additional file 1: Table S1. — Partial correlations of percentage change in renin with biochemical and cardiovascular variables. (DOCX 26 kb) [file 12872_2017_538_MOESM1_ESM.docx]

**Additional file 1: Table S1** Partial correlations of percentage change in renin with biochemical and cardiovascular variables

|  | Percentage change in renin | | | |
| --- | --- | --- | --- | --- |
|  | **Blacks (n=66)** | | **Whites (n=71)** | |
|  | **r-value** | **p-value** | **r-value** | **p-value** |
| Cardiovascular measurements | | | | |
| Systolic blood pressure (%) | **-0.319** | **0.010** | **-0.175** | **0.153** |
| Diastolic blood pressure (%) | **-0.114** | **0.368** | **-0.020** | **0.869** |
| Pulse pressure (%) | **-0.360** | **0.003** | **-0.186** | **0.129** |
| Total peripheral resistance (%) | **-0.079** | **0.531** | **-0.012** | **0.926** |
| Windkessel compliance (%) | **0.105** | **0.409** | **0.047** | **0.704** |
| Carotid intima media thickness (%) | **0.112** | **0.400** | **0.011** | **0.929** |
| Biochemical analyses |  |  |  |  |
| Interleukin-6 (%) | **0.034** | **0.791** | **-0.252** | **0.037** |
| C-reactive protein (%) | **0.078** | **0.539** | **-0.065** | **0.596** |
| Glucose (%) | **0.088** | **0.492** | **-0.214** | **0.077** |
| Von Willebrand factor (%) | **-0.055** | **0.667** | **0.022** | **0.861** |

Adjusted for baseline age and sex
